# Graphene Quantum Dots with Blue and Yellow Luminescence Fabricated by Modulating Intercalation State

**DOI:** 10.3390/ma15196567

**Published:** 2022-09-22

**Authors:** Kwang Hyun Park, Sung Ho Song

**Affiliations:** Division of Advanced Materials Engineering, Center for Advanced Materials and Parts of Powders, Kongju National University, Cheonan-si 31080, Korea

**Keywords:** graphene quantum dot, graphite intercalation compound, photoluminescence, alkali metal, exfoliation

## Abstract

The development of graphene quantum dots (GQDs) with low toxicity, excellent dispersibility, and high photostability has led to extensive progress in bio-imaging and optical sensing applications. However, one-pot synthesis and mass production of GQDs, and tuning their photoluminescence, remains a challenge. Here we demonstrate a simple and scalable method for fabricating GQDs with high size uniformity and chemical stability, via a sequential process of inserting alkali metal into graphite (Stage I: KC_8_ and Stage II: KC_24_) and exfoliation to GQDs in a selected solvent. Structural and optical measurements were conducted, and the emitted colors of the as-prepared GQDs were blue (KC_8_) and yellow (KC_24_), respectively. The stage of graphite intercalation in the compounds played an important role in the size and thickness of the GQD. The as-prepared GQDs had clear characteristic peaks consistent with the quantum confinement effect and intrinsic/extrinsic states. Our approach will provide great potential for a wide variety of bioimaging and bioanalysis applications.

## 1. Introduction

Graphene quantum dots (GQDs), a zero-dimension carbon material, have been intensively studied because they offer low-cost production, high biocompatibility with low toxicity, and fluorescence stability [1,2,3,4]. Also, the fluorescence mechanism of the GQDs, ascribed to quantum confinement and edge effects, permits easy tuning of the band gaps. These excellent properties can be applied in a wide range of fields, including sensing [5,6,7], bioimaging [8,9], solar cells [10,11,12], light-emitting diodes [13,14], and medicine [15].

Efforts to utilize their photoluminescence (PL) properties and various emission colors (blue, green, and red) have been widely reported in studies on GQD synthesis. Among them, the hydrothermal method has been frequently used to fabricate GQDs. Wu’s group reported a GQD with a blue emission, which was fabricated by chemically cutting graphene oxides via a thermal deoxidization process and the resulting GQDs observed a yield of 5% [16]. Chen’s group observed that GQDs synthesized from multi-walled carbon nanotubes (MWCNTs) showed characteristics with a wide size distribution [17]. Unfortunately, the MWCNTs used exhibited swollen and curled features with a partial reaction. Yu’s group used the hydrothermal etching method to prepare graphene quantum dots from single-walled carbon nanotube in HNO_3_, and presented different optical properties because of the degree of oxidation [18]. However, it was difficult to remove excess oxidant from the solution. As the other approaches, Morell’s group demonstrated graphene quantum dots of 2~6 nm size with water-soluble property fabricated by pulsed laser [19]. Recently, Mullen’s group reported the bottom-up synthesis of GQDs with ~60 nm size below 3 nm thickness using cyclodehydrogenation of hexaphenylbenzene [20]. Despite significant advances, these methods still have unresolved issues, such as toxic chemicals and process complexity, and relatively low uniformity of size and thickness.

In this work, we introduce a simple and scalable method for fabricating GQDs with high size-/thickness uniformity and chemical stability via a sequential process. The method involves the insertion of alkali metal into graphite with great stage uniformity (Stage I: KC_8_ and Stage II: KC_24_) and subsequent exfoliation to GQDs in selected solvents. Specifically, potassium metal was spontaneously inserted into graphite crystal, forming potassium graphite intercalation compounds. Also, the intercalation stages can be controlled by adjusting the mixture ratio of graphite and potassium metal, to a highly optimized condition. By precisely controlling the intercalation stages, Stage I (KC_8_) and Stage II (KC_24_) compounds with high crystal uniformity were synthesized, and the compounds were exfoliated into graphene quantum dots with two types of size-/thickness-uniformity. From structural and optical measurements, the as-prepared GQDs showed sizes of ~5 nm with <1.5 nm thickness for Stage I, and ~10 nm with ~3 nm for Stage II. The photoluminescences of Stage I and Stage II GQDs dispersed in distilled water (DI water) had blue and yellow light emissions, respectively. These results indicate that the stage of graphite intercalation compounds plays an important role in the GQD size and thickness as well as photoluminescence (PL). The as-prepared GQDs exhibited clear characteristic peaks of GQDs, which could be attributed to the quantum confinement effect and intrinsic/extrinsic states. The water-soluble GQDs and fabrication system used in this work has great potential for a variety of applications in optoelectronic devices, bioimaging, biosensing, as well as photovoltaic devices as nanophotonic materials [21,22].

## 2. Materials and Methods

### 2.1. Preparation of the GQDs

Graphite powder and potassium metal were used to synthesize different states of potassium graphite intercalation compounds and the compounds were exfoliated to graphene quantum dots (GQDs). Graphite powder and potassium metal were used to synthesize different states of potassium graphite intercalation compounds and the compounds were exfoliated to graphene quantum dots (GQDs). To synthesize potassium graphite intercalation compounds (K-GICs) with different stages, open-end Pyrex tubes with different diameters were used and the diameters for Stage I (KC_8_) and Stage II (KC24) are 6 mm and 12 mm, respectively. The graphite powder (1 g, 0.083 M) was put in Pyrex tubes filled with Potassium metal (0.407 g (0.0104 M) for Stage I (KC_8_) and 0.137 g (0.0035 M) for Stage II (KC_24_). Then the samples were inserted in larger Pyrex tubes (8 mm for Stage I (KC_8_) and 14 mm for Stage II (KC_24_)) with one end open and one closed. After evacuation for 30 min, the samples were stored for 1 h under vacuum in a heating mantle kept at 150 °C. The potassium graphite interaction compounds were put into selected solvents to fabricate the GQDs and treaded for 30 min at 60 °C (DI water and DMSO) and 30 °C (EtOH and Acetone) by tip sonication. DI water is a highly desirable solvent for fabricating GQDs. After purification by dialysis tube, large size graphene sheets were removed by centrifuge at 10,000 rpm. Then, the GQDs of different sizes were obtained and dispersed in the distilled water. The QD solutions were freeze-dried. Finally, GQD powders were obtained.

### 2.2. Characterizations

The size and thickness of the as-prepared GQDs were analyzed by atomic force microscopy (AFM; SPA400; SII, Chiba, Japan) in noncontact mode. The crystallographic structure of the graphite, Stage I (KC_8_), and Stage II (KC_24_) potassium-intercalation compounds structures were measured by X-ray diffraction (XRD; Rigaku Tokyo, Japan). The chemical compositions of the as-prepared GQDs were analyzed by photoelectron spectroscopy (XPS; Sigma Probe; AlKα, Thermo Fisher Scientific, Kyoto, Japan). High-resolution transmission electron microscopy (HR-TEM, Tecnai G2 F30, Hillsboro, OR, USA) was conducted after droplets of the GQDs suspension were deposited on a TEM grid. The photoluminescence (PL) measurements were conducted at room temperature using a 310 nm He-Cd continues-wave (CW) laser, mode-locked femto-second pulsed Ti: sapphire laser (Coherent, Chameleon Ultra II), and monochromatic light from a 300 W Xenon lamp, respectively.

## 3. Results and Discussion

**Figure 1:**
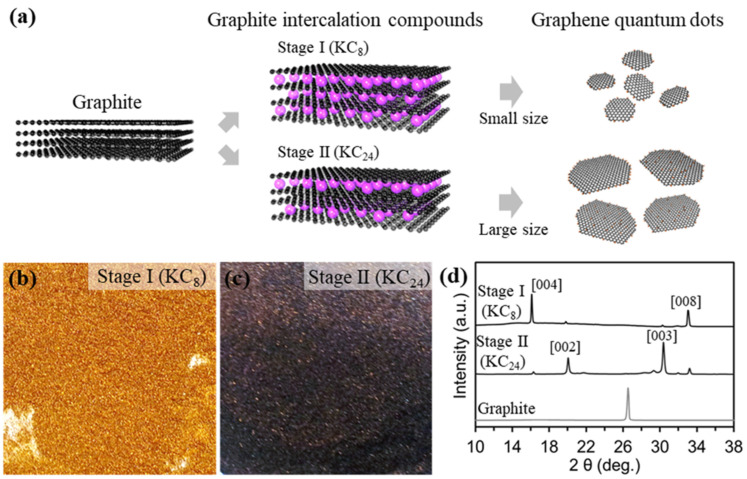
Schematic illustration and characterizations for graphene quantum dots (**a**), fabrication step of GQDs with different sizes QDs. (**b,c**), Digital images of stage I (KC_8_, left) and stage II (KC_24_, right). (**d**), XRD patterns of graphite, KC_8_, and KC_24_.

Figure 1a shows the overall synthetic procedures for fabricating different stages of potassium graphene intercalation compounds, and exfoliation to the size-/thickness-controlled GQDs. The graphite powder (1 g, 0.083 M) was put on potassium metal (0.407 g, 0.0104 M) for Stage I (KC_8_), and potassium metal (0.137 g, 0.0035 M) for Stage II (KC_24_), filled in a Pyrex tube. Samples of Stage I and Stage II mixed in the Pyrex tube were kept at 150 °C for 1 h under vacuum conditions. The molten potassium molecules were spontaneously inserted into the graphite interlayers because of the ionization potential gap [23,24]. Then, the potassium graphite intercalation compounds (K-GICs), KC_8_ with a gold color (Figure 1b) and KC_24_ with a deep blue color (Figure 1c), were exfoliated to GQDs in the selected solvents. Different stages of potassium graphite intercalation compounds were explosively reacted in DI water, EtOH, acetone, and DMSO solvents. To confirm the existence of the GQDs, we conducted photoluminescence measurement at excitation under a 365 nm ultraviolet lamp, as shown in Figure S1. All samples exfoliated in the selected solvents except for DI water showed no significant emitting colors. Also, the emitted color of the Stage I GQD is blue, while the Stage II GQD is yellow. This result suggests that DI water is a highly effective solvent for fabricating the GQDs. these results indicate that the size and thickness of the GQD are influenced by the relation between the K-GIC stages and the selected solvents. The experimental details are further illustrated in the experimental section.

The phase evolution from graphite to Stage I and Stage II was measured by X-ray diffraction (XRD), as shown in Figure 1d. The X-ray diffraction (XRD) patterns revealed the phase transition from graphite to different stages of potassium intercalation compounds (KC_8_ and KC_24_), which is consistent with previous works [24,25,26]. Also, the diffraction peak (d_002_ at 2θ: 26.4) of graphite shifted gradually to lower and higher angles with changes in the potassium intercalation stage. The peak from pristine graphite at 26.4° corresponding to d_002_ was split into four peaks after the potassium intercalation, indicating stage transitions consisting of KC_24_ to KC_8_. Stage II (KC_24_) clearly observed new peaks at 20.1 (d_002_) and 30.4° (d_003_) with small peaks induced from the KC_8_, while Stage I (KC_8_) showed characteristic XRD peaks at 16.9° and 33.4°. These results are comparable with those of KC_8_ and KC_24_ compounds synthesized by two zone vapor-transport and electrochemical intercalation routes [25,26,27,28,29]. Also, colors of the Stage II (KC_24_) and Stage I (KC_8_) were a deep-blue and gold color, which is consistent with previous works [28].

**Figure 2:**
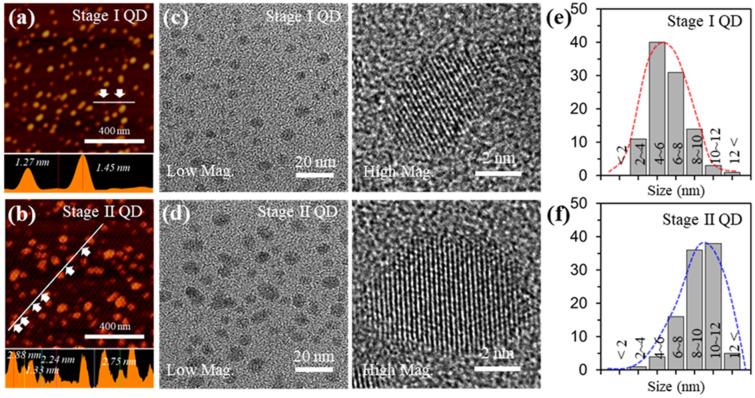
Characterizations for the GQDs with different sizes (**a**,**b**), AFM topology images (Top), and thickness profiles (bottom) of the GQDs. (**c**,**d**), TEM (Left) and HR-TEM (Right) images of the GQDs. (**e**,**f**), size distribution of the GQDs.

Figure 2a,b shows atomic force microscopy (AFM) images of the Stage I QD and Stage II QD and their respective height profiles. The average heights for Stage I QD and Stage II QD were 1.3 ± 0.2 nm and 2.4 ± 0.5 nm, respectively. These results suggest that the as-prepared GQDs mostly consist of less than 10 layers thickness. Figure 2c,d presents transmission electron microscopy (TEM) images of the as-prepared GQDs. The GQDs were uniformly distributed without agglomeration. Also, the shapes of the as-prepared GQDs included a mixture of circular and polygonal shapes. The edges of the GQDs were irregular and unclear because of mixed edges consisting of zigzag and armchair features, which are ascribed to defects and functional groups.

From high resolution (HR)-TEM images (right in Figure 2c,d) the localized lattice fringes of the as-prepared GQDs revealed 0.242 nm corresponding to the (1120) lattice plane and the (002) lattice spacing was 0.34 nm; this is in excellent agreement with what is known for GQDs prepared by other routes [30]. To demonstrate the size uniformity of the as-prepared GQDs, a histogram of the size distribution was prepared by plotting the reprehensive TEM images, as shown in Figure 2e,f. The sizes of the Stage I GQD and Stage II GQD were 4~8 nm (~71%) at 1.3 ± 0.2 nm thickness and 8~12 nm (~74%) at 2.4 ± 0.5 nm thickness, respectively. The average sizes of Stage I GQD and Stage GQD II GQD were ~5 nm and ~10 nm, respectively.

**Figure 3:**
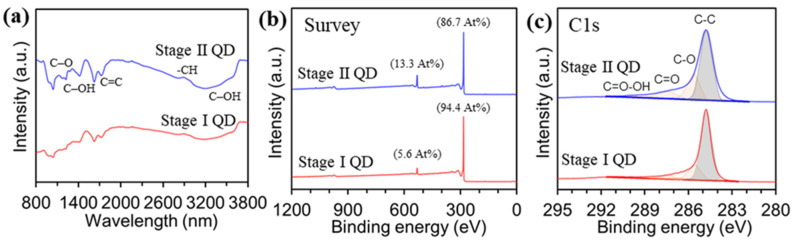
Characterizations for the stage I and stage II GQDs. (**a**), FT-IR spectra. Chemical compositions for carbon and oxygen measured by XPS (**b**), Survey spectra. (**c**), deconvoluted C1s peaks.

The introduction of oxygen or additional elements to the GQDs was evaluated by Fourier-transform infrared spectroscopy (FT-IR) and X-ray photoemission spectroscopy (XPS). Figure 3a shows the chemical bindings of the as-prepared GQDs analyzed by FT-IR measurement. The Stage I GQD exhibited C–O stretching at 1135 cm^−1^, C–OH stretching at 1384 cm^−1^, and 3432 cm^−1^, and C=C stretching at 1632 cm^−1^, and −CH stretching at 2960 cm^−1^, which were similar to the adsorption peaks of the Stage II GQD. The chemical compositions of the Stage I GQD and Stage II GQD were investigated by XPS spectra, to observe the introduction of oxygen or other elements. Figure 3b shows the atomic compositions of carbon and oxygen were 94.4 *At*% and 5.6 *At*% for Stage I GQD, and 86.7 *At*% and 13.3 *At*% for the Stage II GQD, respectively. All of the GQD samples had the C 1s peak with trivial tails in the higher binding energy regions, which can be ascribed to oxygen functional groups, as shown in Figure 3c. To identify functional groups formed on the surface and at the edge of the GQDs, C1s spectra of Stage I and Stage II GQDs were deconvoluted, and the C1s signal consisted of four different peaks: the C–C bond at 284.8 eV, C–O bond at 285.6 eV, C=O bond at 287.8 eV, and O–C=O bond at 289.0 eV.

**Figure 4:**
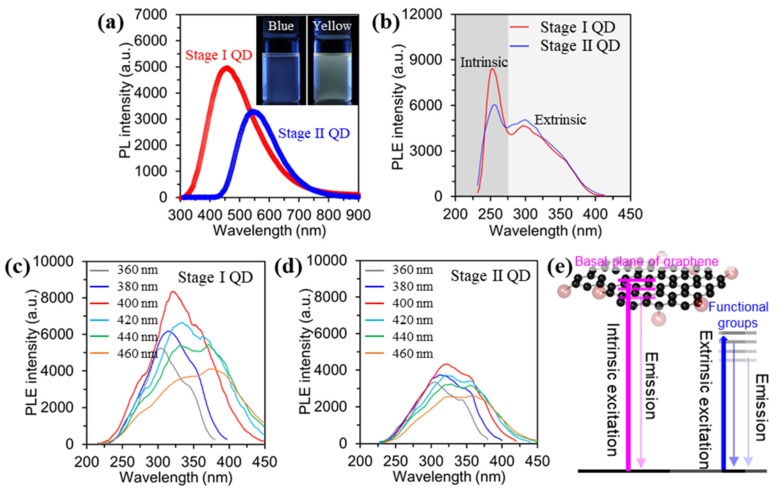
Optical characterizations for the stage I and stage II GQDs. (**a**), PL spectra of the GQDs under excitation at 310 nm. Digital images of the GQDs before and after emission under excitation of λ = 365 nm (Inset). (**b**), PLE spectra of the GQDs. (**c**,**d**), PLE spectra of the GQDs with varying excitation wavelength. (**e**), modeling of the band structure of the as-prepared GQDs based on PL excitation (PLE) data.

Figure 4a shows the PL emission spectra of the as-prepared GQDs dispersed in DI water with excitation from a 310 nm laser. The PL intensity of the Stage I GQD is relatively higher than that of Stage II GQD. Also, the maximum PL positions of the Stage I GQD and Stage II GQD are 450 nm and 550 nm, respectively. The insets in Figure 4a are digital images of the Stage I GQD and Stage II GQD after excitation under a 365 nm ultraviolet lamp. The emitted color of the Stage I GQD is blue, while the Stage II GQD is yellow. These results indicate that the emitting color of the as-prepared GQDs is influenced by the size and thickness of the GQDs, with peak shifting of the maximum PL and PL intensity. UV-vis spectra of the Stage I GQD and Stage II GQD were measured and the result showed in Figure S2. The as-prepared GQD observed two types of shoulder peaks. A shoulder peak at ~260 nm corresponds to the π–π* transition of the aromatic C-C bonds, and a shoulder peak at ~320 nm is assigned to the n-π* of the C=O bonds [16,18]. The band gaps of Stage I and Stage II GQDs were determined from UV-Vis absorption spectra (Figure S3). Furthermore, the quantum yields (QDs) for Stage I and Stage II QDs were measured by using an absolute photoluminescence QY system. The QDs observed at 5.2% (Abs. 0.37%) for Stage I and 3.6% (Abs. 0.33%) for Stage II QD, as shown in Figure S4.

To elucidate the origin of the PL and the carrier dynamics of the GQDs, excitation-wavelength-dependent PL (PLE) were evaluated by manipulating excitation wavelength, as shown in Figure 4b–d. The PLE spectra of the GQDs exhibited a sharp peak at ~ 250 nm, which was ascribed to the π–π* transition in the sp^2^ domain of the GQD (intrinsic states) [31], as shown in Figure 4b. Also, a broad shoulder was observed near 300 nm, related to the n–π* transition of oxygen functional groups, defects, and the varying thickness of the GQDs (extrinsic states) [31]. Overall, the energy position of the Stage I GQD was larger than that of the Stage II GQD because of the PL induced from the intrinsic state, which has higher emission energy than the extrinsic PL. To clarify the characteristics of the extrinsic state of the as-prepared GQDs the PLE spectra were measured by adjusting the excitation wavelength in the range from 360~460 nm, as shown in Figure 3c,d. The Stage I GQDs showed a maximum PLE peak (intrinsic state) at ~320 nm under excitation at 400 nm, and the intensity of the PLE peak gradually decreased with increasing excitation wavelength. Also, the maximum PLE peak (extrinsic state) at ~380 nm was observed under excitation at 420 nm. The overall intensity in the PLE peaks at higher excitation wavelength was decreased. (Figure 4c). Furthermore, Figure 4e showed modeling of the band structure of the as-prepared GQDs based on PL excitation (PLE) data in the range of 360~460 nm (intrinsic state at ~320 nm and extrinsic state at ~380 nm).

On the other hand, the PLE intensity of the Stage II GQD gradually decreased with the red-shifting of the PLE peak position. This result is similar to that of the Stage I GQDs (Figure 4d). As a result, Stage I GQD, which mostly consists of energy levels of the sp2 carbon domain, has intrinsic states, while Stage II GQD revealed extrinsic states with lower energy levels. The Stage I GQD with fewer oxygen functional groups and mono-/bi- layer resulted in the strongest luminescence compared to the Stage II GQDs. In this regard, the PL emission of the Stage II GQD is dominated by extrinsic states, because of a high oxygen content and thickness variation. These optical characteristics of the as-prepared GQDs are well supported by the structural/chemical analysis results (AFM, TEM, FT-IR, and XPS).

## 4. Conclusions

In this work, we demonstrated size-/thickness-controllable GQDs fabricated by the direct exfoliation of potassium graphite interaction compounds designated Stage I and Stage II. The as-prepared GQDs showed excellent dispersion in DI water and strong photoluminescence without chemical additives. Structural/optical analyses indicated the size and thickness of the resulting GQDs were influenced by the stages of the potassium graphite intercalation compound. Furthermore, the optical properties of the GQDs clearly exhibited different characteristics, such as the intensity and position of the photoluminescence, as well as carrier dynamics corresponding to the quantum confinement effect and intrinsic/extrinsic states. The emitting color of Stage I was blue and yellow for Stage II, and was induced by changes in the size and thickness of the GQDs as well as the surface/edge states of the GQDs (especially oxygen functional groups). These results indicate that the water-soluble GQDs in this work have great potential for a variety of applications in optoelectronic devices, bioimaging, biosensing, as well as photovoltaic devices.

## Data Availability

Not applicable.

## Supplementary Materials

The following supporting information can be downloaded at: https://www.mdpi.com/article/10.3390/ma15196567/s1, Figure S1: Dispersion property of Stage I and stage GQD II in the selected solvents; Figure S2: UV-Vis spectra of Stage I QD and Stage II QD; Figure S3: Band gaps of Stage I and Stage II GQDs calculated from the absorption spectra; Figure S4: Quantum yields of Stage I QD and Stage II QD measured by using absolute photoluminescence QY system.
