# Supplementary material for: Graphene Quantum Dots with Blue and Yellow Luminescence Fabricated by Modulating Intercalation State
Source: Materials (Basel). 2022 Sep 22;15(19):6567. doi: 10.3390/ma15196567 (PMC9572107; doi:10.3390/ma15196567)
Supplement: Supplementary file 1 [file materials-15-06567-s001.zip › materials-1888230-supplementary.pdf]

Graphene quantum dots with blue and yellow luminescence fabricated by modulating intercalation state

Kwang Hyun Park, Sung Ho Song *

Division of Advanced Materials Engineering and Center for Advanced Powder Materials and Parts, Kongju National University, Cheonan 32588, Chungnam, Korea

*Correspondence: shsong805@kongju.ac.kr (S.H.S.); Tel.: +82-0415219379 (S.H.S.)

Different stages of potassium graphite intercalation compounds were explosively reacted in DI water, EtOH, acetone, and DMSO solvents. The samples dispersed in the DI water and DMSO treated at 60 °C for 30 min and the samples dispersed in the EtOH and Acetone treated at 30 °C for 30 min by tip sonication.

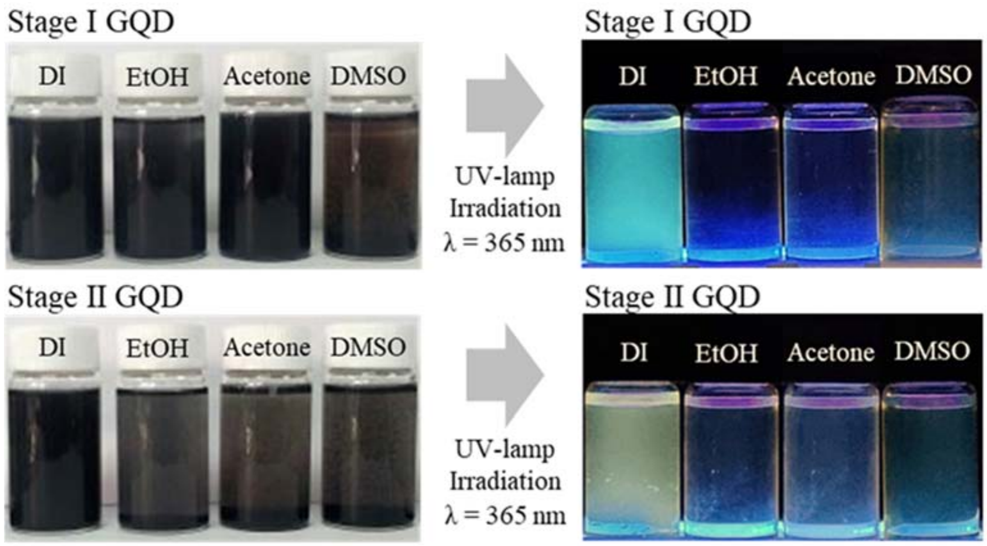

Figure S1. Dispersion property of Stage I and Stage II GQDs in the selected solvents (DI, EtOH, Acetone, DMSO) and Digital images of Stage I and Stage II GQDs in the selected solvents under the excitation of a 365 nm ultraviolet (UV)-lamp.

Two types of shoulder peaks were observed: a shoulder peak at ~ 260 nm corresponds to the π–π* transition of the aromatic C-C bonds, and a shoulder peak at ~ 320 nm assigned to the n-π* of the C = O bonds.

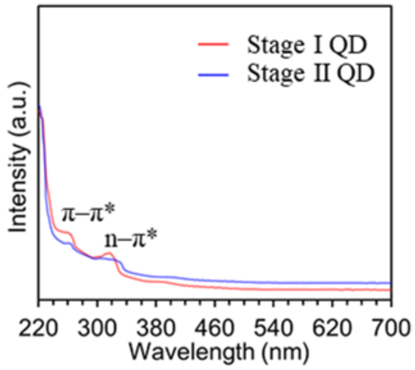

Figure S2. UV-Vis spectra of Stage I QD and Stage II QD.

The band gaps of the Stage I and Stage II GQDs were determined from UV-Vis absorption spectra (Figure S3). Equation (1) is used to calculate the band gaps from the absorption spectra.

$$\alpha hv = (hv - E_g)^2 \qquad \text{Equation (1)}$$

where α = the absorption coefficient, E_g = the bulk band gap energy.

The calculated band gaps of the Stage I and Stage II GQDs were 4.45 eV and 4.04 eV, respectively.

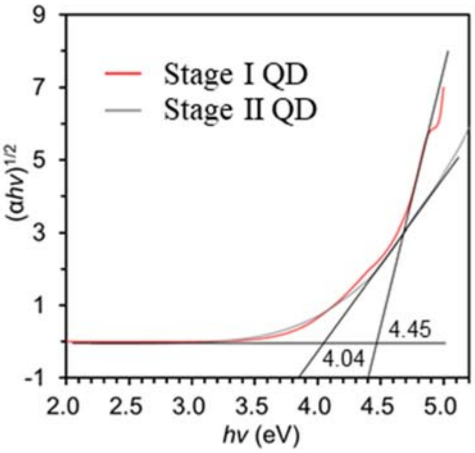

Figure S3. Band gaps of Stage I and Stage II GQDs calculated from the absorption spectra

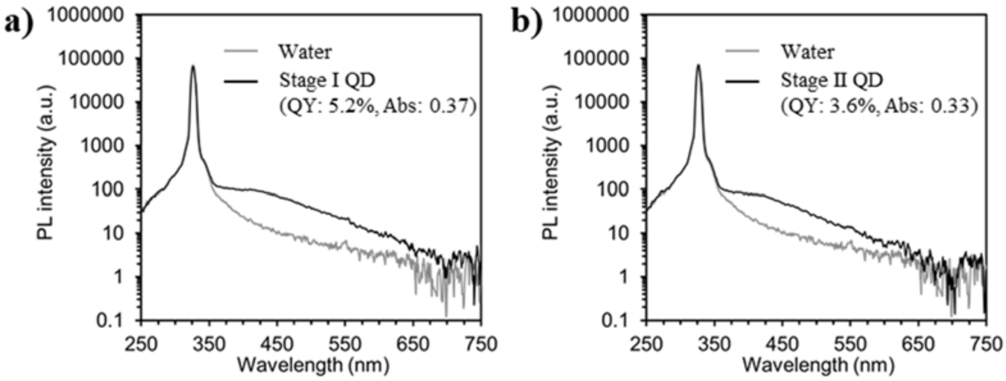

Figure S4. Quantum yields of Stage I QD and Stage II QD measured by using absolute photoluminescence QY system.
